# Isolation and Characterization of Human Lung Lymphatic Endothelial Cells

**DOI:** 10.1155/2015/747864

**Published:** 2015-06-07

**Authors:** Bruno Lorusso, Angela Falco, Denise Madeddu, Caterina Frati, Stefano Cavalli, Gallia Graiani, Andrea Gervasi, Laura Rinaldi, Costanza Lagrasta, Davide Maselli, Letizia Gnetti, Enrico M. Silini, Eugenio Quaini, Luca Ampollini, Paolo Carbognani, Federico Quaini

**Affiliations:** ^1^Department of Clinical and Experimental Medicine, University Hospital of Parma, 43126 Parma, Italy; ^2^Department of Biomedical, Biotechnological, and Translational Sciences (S.Bi.Bi.T.), University Hospital of Parma, 43126 Parma, Italy; ^3^Cardiovascular Department, Humanitas Clinical and Research Centre, 20089 Milan, Italy; ^4^Department of Surgical Sciences, University Hospital of Parma, 43126 Parma, Italy

## Abstract

Characterization of lymphatic endothelial cells from the respiratory system may be crucial to investigate the role of the lymphatic system in the normal and diseased lung. We describe a simple and inexpensive method to harvest, isolate, and expand lymphatic endothelial cells from the human lung (HL-LECs). Fifty-five samples of healthy lung selected from patients undergoing lobectomy were studied. A two-step purification tool, based on paramagnetic sorting with monoclonal antibodies to CD31 and Podoplanin, was employed to select a pure population of HL-LECs. The purity of HL-LECs was assessed by morphologic criteria, immunocytochemistry, flow cytometry, and functional assays. Interestingly, these cells retain *in vitro* several receptor tyrosine kinases (RTKs) implicated in cell survival and proliferation. HL-LECs represent a clinically relevant cellular substrate to study lymphatic biology, lymphoangiogenesis, interaction with microbial agents, wound healing, and anticancer therapy.

## 1. Introduction

The lymphatic system consists of a vascular network of blind-end, thin-walled capillaries and larger vessels that drain lymph, from the extracellular spaces of most organs, to the venous circulation through the thoracic duct. The lymphatic capillary wall is composed of a continuous single-cell layer of overlapped endothelial cells. Due to the discontinuous basement membrane, these vessels are highly permeable. The lymphatic system also includes lymphoid organs such as lymph nodes, tonsils, Payer's patches, spleen, and thymus. Lymphatic vessels are usually absent in avascular structures and in some vascularized organs such as brain, retina, and bone marrow. By regulating tissue fluid homeostasis, immune cell trafficking, and dietary fats absorption the lymphatic system plays a pivotal role in inflammation, wound healing, and tumour metastasis [1–4]. Recently, a murine model has been proposed in which pulmonary lymphatic function is required in late fetal life to prevent tissue edema and ensuring lung compliance at birth [5].

In the last fifteen years, an important aid in some aspects of* in vitro* and* in vivo* vascular biology has been prompted by the identification of lymphatic markers that can accurately discriminate lymphatic vessels from blood vessels. Thus, hyaluronan receptor Lyve-1 [6], the prospero-related homeobox gene transcription factor Prox1 [7], the membrane glycoprotein podoplanin (Pdn)/D2-40 [8], vascular endothelial growth factor receptor- (VEGFR-) 3 tyrosine kinase [9], and its ligands VEGF-C and VEGF-D [10] have been documented in the specification and lineage commitment of the lymphatic system.

Evidence has also been provided that specific congenital or acquired disorders of the lymphatic system may lead to pathologic conditions such as lymphangitis, lymphoedema, lymphangioma, and lymphangiosarcoma [11].

Although lymphatics in the lung have been described over centuries ago [12] detailed reports on the precise identification of human lung lymphatics are at best sporadic.

Histologic characterization of pulmonary structures showed that lymphatic vessels are located in close proximity of the airways and major blood vessels, a phenomenon likely related to their role in alveolar clearance necessary to obtain an efficient respiration. Although not shared by experts in the field, according to several investigators, this specific function sees the lymphatic contribution of greater importance than that of blood vasculature [13–16]. In this regard, the low numerical incidence and flattening of lymphatics make it difficult to assess their morphometric evaluation in interalveolar septa of the normal human lung and therefore possible functional correlations [13, 15]. However, by immunohistochemistry [13] or cast scanning electron microscopy [15], measurable quantity of lymphatics mainly located in the interlobular and bronchovascular interstitium seems to be less than half of that of blood vessels [13]. Interestingly, an increased density and surface area of lymphatics was observed in the peribronchiolar space as compared to perivascular location [13].

Although lymphatics and lymphangiogenesis have been implicated in the pathogenesis of lung diseases, the assessment of their biologic and functional involvement is missing. Human studies have shown* de novo* lymphangiogenesis or lack of lymphatics, respectively, in patients affected by diffuse alveolar damage or severe asthma [17–19]. In idiopathic pulmonary fibrosis a severe injury of subpleural and interlobular lymphatics suggested alveolar clearance as a new putative pathogenetic mechanism of the disease [20, 21]. Structural and functional changes in the lymphatic vascular system have also been implicated in the setting of lung transplantation and allograft rejection [11].

It is clear that a better understanding of organ specific lymphatics may lead to identify cellular and molecular targets, whose modulation may interfere with several pathologic processes including neo-lymphangiogenesis induced by tumours and their metastatic diffusion.

Cell culture models may be crucial to elucidate the pathobiology of lung microvascular endothelium. However, most of our current knowledge is derived from experiments on cultured human umbilical cord vein endothelial cells (HUVECs).

Methods to obtain lymphatic endothelial cells from several tissues [22–25] have been reported and different isolation procedures have been proposed to isolate a relative pure population of pulmonary microvascular endothelial cells from humans and murine tissues [26–30]. Yet, a detailed description of protocols to harvest, culturing and propagate lymphatic endothelial cells (LECs) from the human lung has not been published.

Here, we describe a simple and inexpensive method, requiring minimum equipment and accessories, to obtain lymphatic (HL-LECs) and blood (HL-BECs) endothelial cells from healthy human lung using a two-step purification tool based on sequential sorting with monoclonal antibodies to CD31- and Pdn-coated paramagnetic beads.

HL-LECs were extensively characterized by morphology, including transmission electron microscopy (TEM), immunocytochemistry, and flow cytometry, and their ability to form tube-like structures on Matrigel was assessed.

## 2. Materials and Methods

### 2.1. Tissue Sampling

The study was performed on 55 patients affected by lung cancer and undergoing lobectomy or pneumonectomy at the Department of Surgical Sciences, University-Hospital of Parma. Patients were enrolled after informed consent to the employment of biologic samples for research purpose. The procedure was approved by the institutional review board for human studies (Ethical Committee) of the University-Hospital of Parma and in accord with principles listed in the Helsinki declaration.

Lung tissue was collected and transferred under sterile condition to the Laboratory of Pathology (S.Bi.Bi.T. Dept). Samples were processed by the medical staff ensuring the priority of their use for diagnostic purposes within 1 hour from resection. Fragments of 0.5–1 g were obtained at safety distance of minimum 7 cm from the tumour. The correct sampling procedure to exclude pathologic findings was carried out by subsequent histologic analysis.

Fresh, healthy lung tissues were shortly immersed in sterile PBS to perform cell isolation or fixed in 10% neutral buffered formalin and embedded in paraffin for histologic and immunohistochemical analysis.

### 2.2. Immunohistochemistry

To ensure that harvested pulmonary tissues did not contain tumour lesion or other pathologic conditions, fixed samples were stained with hematoxylin and eosin (H&E). Immunohistochemical detection of lymphatic vessels was performed on paraffin-embedded sections cut at 5 *μ*m thickness. After deparaffinization, heat-induced antigen retrieval was carried out in 10 mM sodium citrate buffer (pH 6) for 15 minutes by microwave oven. Sections were cooled down to room temperature (RT), washed in distilled water, rinsed in PBS and incubated with 20% goat serum (Sigma Aldrich, St. Louis, MO, USA) for 30 minutes at RT to block unspecific binding.

Sections were incubated with primary antibodies: mouse anti-human CD31 (ready to use; overnight (o/n) 4°C; DAKO, Copenhagen, Denmark) and rabbit anti-human Pdn (1 : 100; o/n 4°C; ReliaTECH, Braunschweig, Germany). TRITC- and FITC-conjugated anti-mouse and anti-rabbit antibodies (Sigma Aldrich), respectively, were used to detect simultaneously the two epitopes. Nuclei were counterstained with 0.5 mg/mL 4′,6-Diamidino-2-phenylindole (DAPI; 18 minutes RT; Sigma Aldrich). Slides were mounted by Vectashield (Vector Laboratories, Burlingame, CA, USA) and analyzed using a fluorescence Microscope (Leica DMI6000B, Heidelberg, Germany). Fluorescent images were digitally captured with “LAS Advanced Fluorescence” software (Leica) connected to the microscope provided by a digital camera (Leica DFC350FX).

### 2.3. Processing of Fresh Lung Tissue

Fragments of healthy lung specimens were washed several times with phosphate buffer solution (PBS) and finely minced with scissors and subjected to enzymatic digestion for 75 minutes at 37°C with 1 mg/mL collagenase/dispase solution (Roche, Basel, Switzerland). The resulting digestion product was squeezed over a 100 *μ*m nylon mesh (BD Biosciences, San Josè, CA, USA) to remove aggregates. The harvested cells were washed, seeded on collagen coated wells of a 6-well plate (BD Biosciences) and cultured in complete growth medium consisting of endothelial growth medium-MV (EGM-MV; Lonza, Walkersville, MD, USA) plus 5% fetal bovine serum (FBS; Lonza) and 2 ng/mL recombinant human vascular endothelial growth factor-C (VEGF-C; ReliaTECH).

Twenty-four hours later, nonadherent cells and debris were removed, whereas the adherent cell population was washed with PBS and cultured in complete medium until 70–80% confluence (usually reached in 5–7 days).

### 2.4. LEC Isolation and Culture

Primary cultures were harvested by trypsin/EDTA (Sigma Aldrich), incubated with 20 *μ*L FcR reagent and 20 *μ*L anti-human CD31 immunolabeled magnetic microbeads, according to manufacturer's recommendations (CD31 Microbeads Kit; Miltenyi Biotec, Bergisch Gladbach, Germany).

In brief, after 15 minutes at 4°C, cells were loaded onto a MS Column (Miltenyi Biotec) and placed in the magnetic field of an OctoMACS Separator (Miltenyi Biotec). All recovered ECs, positively selected for CD31 by the magnetic particle concentrator, were cultured on collagen-coated T25 flasks (BD Biosciences) in complete medium.

When reached 80–90% of confluence, CD31 selected cells were detached by trypsinization, washed, resuspended in 100 *μ*L PBS, and incubated, for 20 minutes at 4°C, with 1 *μ*g mouse anti-human Pdn (ReliaTECH). After incubation, cells were washed, centrifuged, resuspended in 30 *μ*L PBS 0.5% FBS, and incubated with 15 *μ*L goat anti-mouse antibodies-coated magnetic microbeads (MiltenyiBiotec) for 15 minutes at 4°C. Cells were reloaded onto a MS Column placed in the magnetic field of an OctoMACS Separator and eluted with 2 mL PBS added with 0.5% FBS. The unsorted population, being the CD31^pos^/Pdn^neg^ cell fraction and magnetically labelled Pdn^pos^ ECs retained on the column were separately processed. After removal of column from the magnetic field, the magnetically retained Pdn sorted cells were eluted with 2 mL PBS containing 0.5% FBS and represented Lymphatic Endothelial Cells (LECs). LECs were seeded, cultured, and propagated with EGM-MV plus 5% FBS and 2 ng/mL VEGF-C. Pdn unsorted cells representing Blood Endothelial Cells (BECs) were seeded, cultured, and propagated with EGM-MV plus 5% FBS.

Human lung lymphatic endothelial cells (HL-LECs) and human lung blood endothelial cells (HL-BECs), routinely examined by inverted light microscopy (Olympus CK40, Tokyo, Japan), were serially sub-cultured at a split ratio of 1 : 5 onto biocoat T25 flask (1 × 10^4^ cells/cm^2^). HL-LEC could be easily expanded from passage 2 (P2) to 8 (P8) without changes in their morphology or evidence of cellular senescence. Several cryogenic vials (VWR International PBI, Radnor, PA, USA) containing 5 × 10^5^ endothelial cells from each recovered cell lines were frozen in cryopreservation solution containing 90% (v/v) FBS (Sigma Aldrich) and 10% (v/v) dimethyl sulfoxide (Sigma Aldrich) for storage in liquid nitrogen and to create a master cell bank. The vials were placed overnight in a cryo 1°C freezing container (VWR International PBI) at −80°C and transferred after 24–48 hours in liquid nitrogen. All the experiments described here were performed on HL-LECs from P2 to P8.

### 2.5. TEM Analysis

HL-LECs were analyzed by transmission electron microscopy (TEM) to detect the typical subcellular features associated with the lymphatic endothelium.

HL-LECs were expanded and after trypsinization centrifuged to obtain a visible pellet. The pellet was fixed in Karnovsky solution (4% formaldehyde, 5% glutaraldehyde) for 90 minutes at RT After washing with phosphate buffer, pH 7.2, the pellet was embedded in agar to maintain cohesiveness for subsequent processing.

Sample were post-fixed in 1% osmium tetroxide (OsO_4_) for 90 minutes at RT and dehydrated by increasing concentration of alcohol. Following this procedure, samples were washed with propylene oxide and embedded in epoxy resin (Durcupan ACM, Sigma Aldrich). After resin polymerization, samples were trimmed on sections of 0.5 *μ*m thickness and subsequently stained with methylene blue and safranin to select morphologically the field of interest. Ultrathin sections were collected on a 300-mesh copper grid and, after staining with uranyl acetate and lead citrate, were qualitatively examined under a transmission electron microscope (Philips EM 208S).

### 2.6. Flow Cytometric Analysis

HL-LEC confluent monolayers at P3 and P8 were dissociated with trypsin/EDTA, the cell pellet resuspended in PBS with 1% FBS and aliquot at 1 × 10^5^ cell/tube for antibody labeling. One hundred microlitres of the cell suspension were incubated for 30 minutes at 4°C with 1 *μ*g mouse anti-human Pdn (ReliaTECH). Primary antibody binding was detected by goat anti-mouse IgG FITC. Negative controls were represented by omission of the primary antibody from the reaction. Live cells were gated using forward (FSC) versus side scatter (SSC) dot plot.

Cells were analyzed by a FACS-Diva software (FACScanto II, BD Biosciences) acquiring at least 50,000 events.

### 2.7. Immunocytochemistry

Immunocytochemistry was performed on HL-LEC cultured on chamber slides (BD Biosciences) pretreated with collagen solution type 1 (Sigma Aldrich). When 80% confluence was reached, cells were fixed in cold 4% paraformaldehyde in PBS, pH = 7.4 for 30 minutes at RT. After washing with PBS, HL-LECs were exposed to 20% goat serum (Sigma Aldrich) for 30 minutes at RT to block unspecific binding.

Cells were then incubated with the following primary unconjugated antibodies: mouse anti-human CD31 (ready to use; o/n 4°C; DAKO), rabbit anti-human von Willebrand Factor (vWF) (1 : 200; o/n 4°C; DAKO), rabbit anti-human Prox-1 (1 : 20; 75 minutes 37°C; Acris, Herford, Germany) after light permeabilization with 0.1% Triton X100, mouse anti-human alpha-smooth muscle actin (*α*-SMA) (1 : 200; o/n 4°C; DAKO), mouse anti-human vascular endothelial growth factor receptor-2 (VEGFR-2/KDR) (1 : 100; o/n 4°C; ReliaTECH), rabbit anti-human VEGFR-3 (1 : 25; o/n 4°C; Abcam). Fluorochrome-conjugated secondary antibodies (1 : 70; 60 minutes 37°C; Sigma Aldrich) were employed to detect different epitopes by fluorescence microscopy. Nuclei were recognized by counterstaining with DAPI for 15 minutes at RT and cover slips mounted with Vectashield.

Samples were analyzed using a fluorescence microscope (DMI6000B Leica). Fluorescent images were digitally captured with “LAS Advanced Fluorescence” software (Leica) connected to a microscope provided by a digital camera (Leica DFC350FX).

D2-40, LYVE-1, Fibroblast Growth Factor Receptor (FGFR)-1, Epidermal Growth Factor Receptor (EGFR), hepatocyte growth factor receptor c-MET, type 1 Insulin-like Growth Factor Receptor (IGF-1R), Platelet-Derived Growth Factor Receptor (PDGFR)-beta, Tropomyosin-related kinases A (TrkA), and neurotrophin p75 receptor (p75^NTR^) were investigated by immunoperoxidase. Briefly, after fixation, chamber slides of HL-LECs were immersed in 3% hydrogen peroxide solution for 10 minutes and then incubated with mouse anti-human D2-40 (1 : 50; o/n 4°C; Biocare Medical, Concord, CA, USA), rabbit anti-human LYVE-1 (1 : 50; o/n 4°C; Abcam, Cambridge, UK), rabbit anti-human FGFR-1 (1 : 40; o/n 4°C; Cell Signaling, Beverly, MA, USA), mouse anti-human EGFR (1 : 40; o/n 4°C; Zymed-Invitrogen, Grand Island, NY, USA), rabbit anti-human c-MET (according to manufacturer's recommendations; Ventana-Roche), rabbit anti-human IGF-1R (1 : 30; o/n 4°C; Cell Signaling), rabbit anti-human PDGFR-beta (1 : 25; o/n 4°C; Abcam), rabbit anti-human TrkA (1 : 40; o/n 4°C; Santa Cruz Biotechnology, Heidelberg, Germany), and rabbit anti-human p75^NTR^ (1 : 70; o/n 4°C; Millipore, Darmstadt, Germany), respectively. The immunoreaction was revealed by anti-mouse and anti-rabbit secondary antibodies polymerized with horseradish peroxidase in Tris-HCl buffer following the instruction of the advance/HRP kit (DAKO). Cytologic preparations were then counterstained with hematoxylin and analysed by an optical microscope (Olympus BX60) connected to QICAM camera (QImaging, Surrey, BC, Canada).

For each epitope tested by either immunoperoxidase or immunofluorescence, negative controls were represented by samples incubated with secondary antibodies and omitting the primary antibodies from the reaction. The specificity of the immune reaction was documented by exposing our endothelial cell preparations to antibodies to CD45 used as an unrelated antigen (see Figure S4 in Supplementary Material available online at http://dx.doi.org/10.1155/2015/747864). Moreover, human dermal fibroblasts were employed as negative controls for the immunocytochemical detection of endothelial markers.

### 2.8. *In Vitro* Angiogenesis Assay

The tube formation assay was performed according to manufacturer's recommendations and previously reported methods with some modifications [31]. Briefly, 80 *μ*L of Matrigel (BD Biosciences) were loaded at the bottom of well of the 96 well plates (BD Biosciences). After 30 minutes at 37°C, cells (2 × 10^4^ cells/100 *μ*L in each well) suspended in complete growth medium were gently layered on the top of Matrigel. After 24 hours, to assess tube-forming ability, images were captured using an inverted microscope (Olympus CK40) connected to a digital camera (Olympus DP21).

## 3. Results

### 3.1. Immunohistochemical Detection of Human Lung Lymphatics

A fragment of lung samples utilized for cell isolation was subjected to microscopic examination. Sections stained with H&E were analysed to exclude pathologic findings and to identify areas of preserved alveolar parenchyma (supplemental data, figure S1A). To detect lymphatic vessels, double immunolabelling with CD31 and the lymphatic marker Pdn was performed.

In agreement with previous studies [13–16], lymphatic vessels in healthy lung tissues were documented by the presence of CD31^pos^/Pdn^pos^ vascular profiles (supplemental data, figure S1B). Blood endothelial cells were labeled by CD31 only (supplemental data, figure S1B).

### 3.2. Isolation and Culture of HL-LEC and HL-BEC

**Figure 1:**
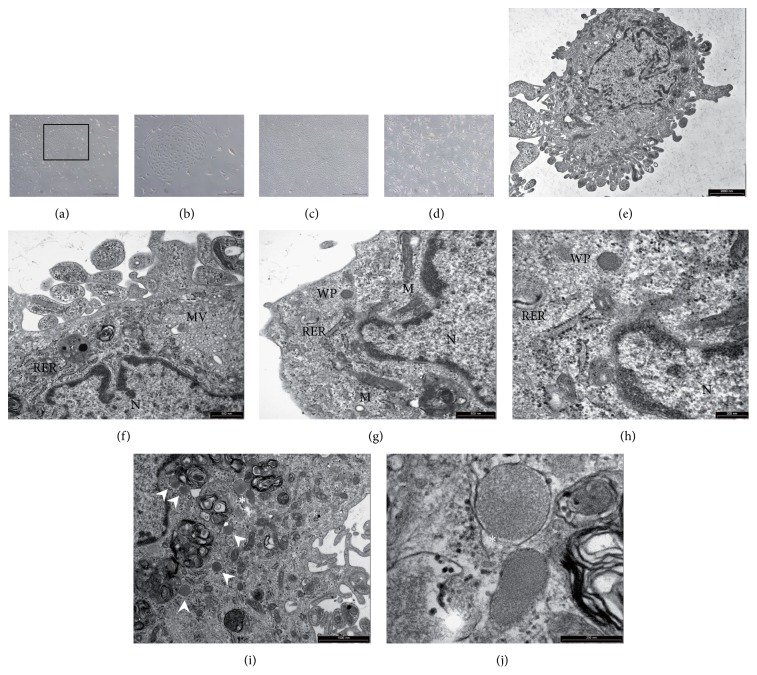
Morphology of human lung lymphatic endothelial cells (HL-LEC). (a)–(d): phase contrast microscopy. (a): an example of a mixed cell population obtained after enzymatic digestion of lung fragments. The black rectangle includes an area shown at higher magnification in (b) to illustrate a cluster of cells with cobblestone-like morphology, suggestive of an endothelial phenotype. (c): confluent monolayer of lymphatic endothelial cells selected by double immunomagnetic sorting with CD31 and Pdn antibodies. (d): fibroblastoid morphology of the CD31 negative cell population eluted after immunomagnetic sorting. (e)–(j): Transmission Electron Microscopy (TEM). (e): microphotograph of a single HL-LEC showing numerous surface microvilli. (f)–(h): ultrastructural characteristics of the lymphatic endothelium are documented by the presence of prominent micropinocytotic vesicles (MV), a well-developed rough endoplasmic reticulum (RER) and Weibel-Palade bodies (WP). (i)-(j): ultrastuctural image of a HL-LEC showing multiple Weibel-Palade bodies (white arrowheads) Two adjacent WPBs (asterisk), one transversally and one obliquely oriented, are shown at higher magnification in (j) to appreciate microtubular-like structures. M: mitochondria. N: nucleus. Scale bars: (a), (c), and (d) = 500 *μ*m; (b) = 200 *μ*m; (e) = 2 *μ*m; (f) and (g) = 0.5 *μ*m; (h) and (j) = 0.2 *μ*m; (i) = 1 *μ*m.

After tissue digestion, primary cultures of a heterogeneous adherent cell population were obtained, mainly consisting of stromal cells, fibroblasts, macrophages and endothelial cell clusters at time displaying characteristic “cobblestone-like” morphology (Figures 1(a) and 1(b)). Sub-confluent cell monolayers were recovered with trypsin/EDTA and sorted with microbeads coated with anti-CD31 antibodies to remove contaminating cells (Figure 1(d)).

CD31 sorted ECs were cultured until confluence recovered with trypsin/EDTA and additionally sorted with anti-Pdn immunolabelled microbeads. By this approach, highly purified LECs from human lung tissue (HL-LEC) were successfully harvested (Figure 1(c)) and further expanded for their characterization (see below).

The negative selection from Pdn sorting was recovered and represented blood endothelial cells (BECs). Although also BECs were seeded, cultured, and propagated onto biocoat flask with EGM-MV plus 5% FBS, this cell population will not be detailed in our report.

This approach allowed us to isolate endothelial cells from 51 of the 55 processed human lung samples with a yield of 92%.

### 3.3. HL-LECs Propagation and Cryopreservation

HL-LECs were seeded, cultured and propagated (ratio 1 : 5) onto biocoat T25 flask (1 × 10^4^ cells/cm^2^) with EGM-MV plus 5% FBS and 2 ng/mL VEGF-C for at least 8 passages without significant changes in morphology, growth properties and evidence of cellular senescence (data not shown). A uniform monolayer was initially identified as endothelial cells according to morphologic criteria. In typical cultures, from P2 to P8, the doubling time of HL-LEC was 32.3 ± 2.5 hours.

At several passages, cells were aliquoted and cryopreserved. Subsequently, random cryogenic vials were thawed with a good recovery (data not shown).

### 3.4. TEM Analysis

The typical ultrastructural features of the lymphatic endothelium were documented by TEM. Lung derived cultured endothelial cells showed prominent microvilli on the cell surface (Figure 1(e)). In addition to the presence of abundant cytoplasmic microvescicles and a well-developed rough endoplasmic reticulum (Figure 1(f)), electron-dense rod-shaped microtubulated bodies, originally described by Weibel and Palade [32], were detected in cultured HL-LECs (Figures 1(g)–1(j)). Moreover, as previously reported [33], numerous autophagic vacuoles and parallel aggregates of intermediate filaments were frequently observed in expanded endothelial cells (data not shown).

### 3.5. Immunophenotypic Characterization of HL-LECs

#### 3.5.1. Flow Cytometric Analysis

**Figure 2:**
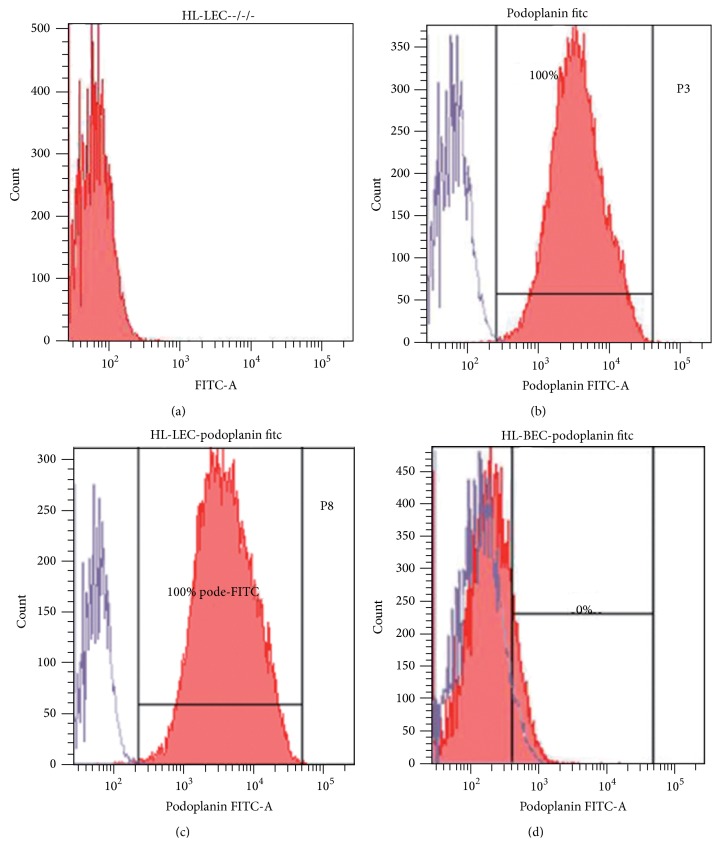
Fluorescence activated cell sorting. (a)–(c): Representative flow cytometric assay of lymphatic endothelial cells from the human lung (HL-LEC) expanded after double immunomagnetic sorting. (a): negative control represented by the omission of primary antibodies. (b) and (c): virtually 100% of HL-LECs show strong specific positivity for antibodies against Pdn both at passage 3 (P3, (b)) and 8 (P8, (c)). (d): FACS plot of HL-BECs eluted from Pdn immunomagnetic sorting (P4).

To ascertain the efficiency in cell separation and the lymphatic nature of HL-LECs, FACS analysis of the expression of Pdn was performed. As shown in Figures 2(b) and 2(c), 100% of isolated and cultured lymphatic endothelial cell lines from human lung tissues were positive for the lymphatic specific marker at P3 and this property was preserved until P8. Cells eluted from Pdn immunomagnetic sorting representing HL-BECs did not express the lymphatic associated antigen at FACS analysis (Figure 2(d)).

#### 3.5.2. Immunocytochemical Analysis

To ensure the purity of cells recovered by double immunomagnetic sorting, immunocytochemistry was performed on LECs cultured on chamber slides.

**Figure 3:**
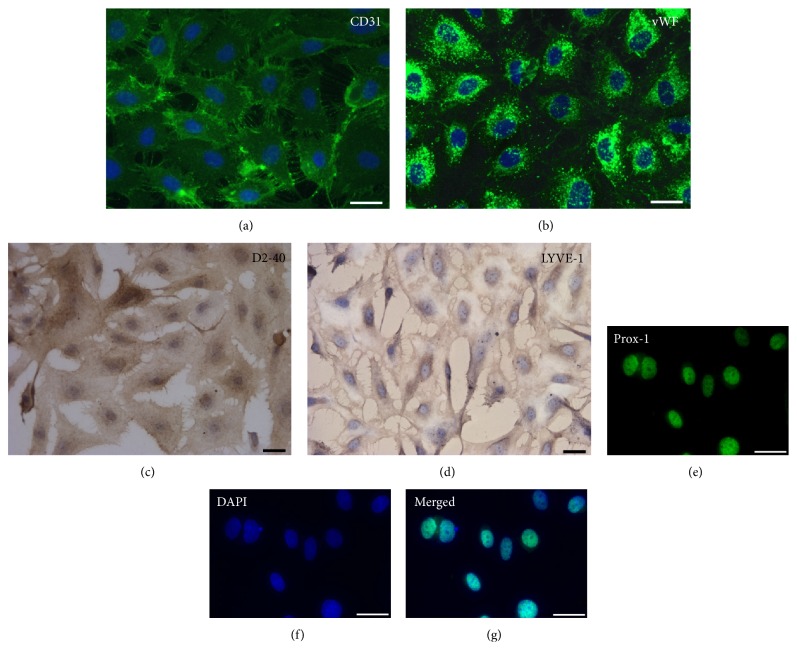
Immunocytochemical characterization of cultured human lung endothelial cells (HL-LECs). (a) and (b): the surface expression of the pan-endothelial marker CD31 and the dot-like cytoplasmic abundance of von Willebrand Factor (vWF) are shown in green by immunofluorescence. (c) and (d): immunoperoxidase detection of lymphatic associated antigens D2-40 and LYVE-1 (brownish) in HL-LEC. (e)–(g): the specific lymphatic lineage commitment of HL-LECs is documented by the nuclear expression of Prox-1 transcription factor (green). Nuclei are counterstained by DAPI in (a), (b), (f) and (g) and by Haematoxylin in (c) and (d). The immunocytochemical analysis was conducted on HL-LECs at passage 4 (P4). Scale bars: (a)–(g) = 25 *μ*m.

The pan-endothelial marker CD31 is an integral membrane glycoprotein, also known as Platelet Endothelial Adhesion Molecule-1 (PECAM-1), which is constitutively expressed on the surface of mature endothelial cells. CD31 is highly enriched at junctions between adjacent endothelial cells, both* in vitro* and* in vivo* [34, 35]. Accordingly, virtually all our cultured LECs were positive for CD31 with abundant membrane expression (Figure 3(a)). Similarly, von Willebrand factor (vWF) glycoprotein, exhibited as strong granular cytoplasmic expression (Figure 3(b)). Interestingly, vWF is stored in endothelial cell in Weibel-Palade bodies [32] further strengthening their detection by TEM. In contrast, the expression of *α*-SMA was constantly absent in our preparation of HL-LEC (data not shown).

To define the lymphatic phenotype, three specific cell markers D2-40, LYVE-1 and Prox-1 were examined.

D2-40/podoplanin is a mucin-type transmembrane glycoprotein and unlike CD31, which recognizes both blood and lymphatic endothelial epitopes, its immunoreactivity is restricted and selectively expressed in LEC. Results of our investigation documented that all LECs lines obtained from the human lung expressed Pdn at a rate of 100% (Figure 3(c)).

LYVE-1, the lymphatic receptor for the extracellular matrix mucopolysaccharide hyaluronan, was widely expressed in our preparations of LECs showing a characteristic pattern on cell membrane surface (Figure 3(d)).

Prox-1, a key control gene in the program that specifies lymphatic endothelial cell fate, was consistently expressed by lymphatic endothelial cells isolated from healthy human lung tissue (Figures 3(e), 3(f), and 3(g)).

Importantly, the expression of these markers was preserved in cells expanded at least for 8 passages.

Thus, by immunocytochemistry, our population of HL-LEC possesses the distinct phenotypic characteristics of the lymphatic endothelium.

### 3.6. HL-LECs Express Multiple Tyrosine-Kinase Receptors

Receptor tyrosine kinases (RTKs) are transmembrane receptors that phosphorylate tyrosine residues with specificity in various protein substrates. RTKs play an important role in cell signaling and are involved in a variety of processes. In particular, in lymphatic endothelial cells RTKs are highly relevant due to their essential role in cell survival, proliferation, migration, and lymph-angiogenesis [36].

Thus, the presence of TK receptors in our primary cultures of human HL-LECs was investigated.

**Figure 4:**
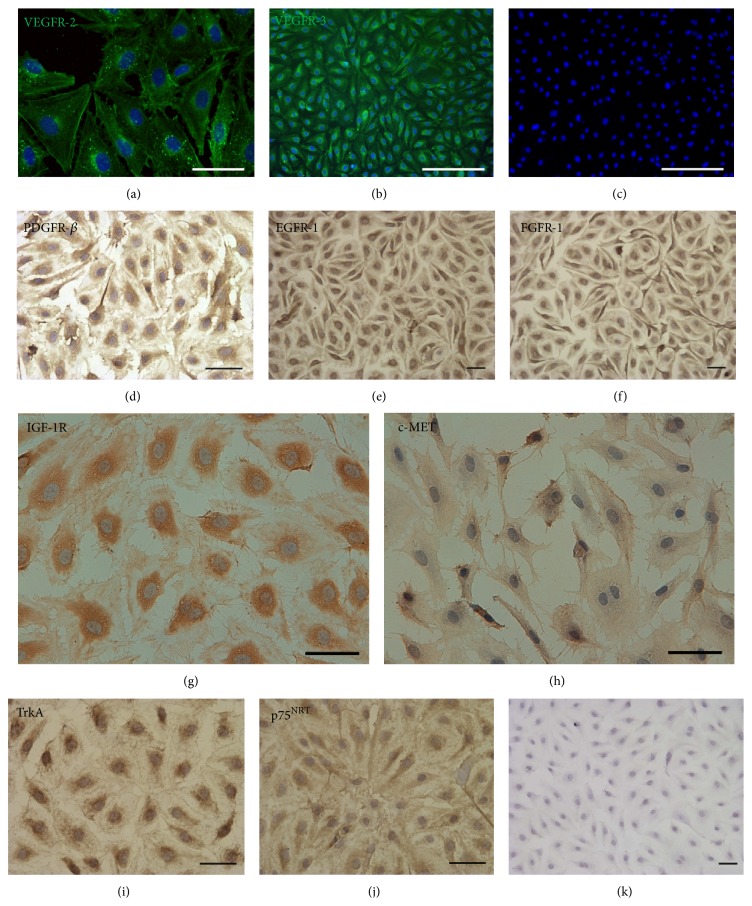
Detection of Receptor Tyrosine Kinases on HL-LECs (P4). (a) and (b) (immunofluorescence): green fluorescence corresponds to the surface expression of vascular endothelial growth factor receptor- (VEGFR-) 2 and VEGFR-3, respectively, in cultured HL-LECs. (c): representative image of a negative control in which the primary antibody was omitted from the reaction. Nuclei are shown by the blue fluorescence of DAPI. (d)–(j) (immunoperoxidase): the brownish precipitate on the surface of HL-LECs on each panel corresponds respectively to the expression of Platelet-Derived Growth Factor Receptor- (PDGFR-) beta, Epidermal Growth Factor Receptor- (EGFR-) 1, Fibroblast Growth Factor Receptor- (FGFR-) 1, type 1 Insulin-like Growth Factor Receptor (IGF-1R), the Hepatocyte Growth Factor receptor c-MET, Tropomyosin-related kinases A (TrkA), and neurotrophin p75 receptor (p75^NTR^). (k): representative image of a negative control in which the primary antibody was omitted from the reaction. Nuclei are counterstained with Haematoxylin. Scale bars: (a), (d), (e), (f), (g), (h), (i), (j), and (k) = 50 *μ*m; (b) and (c) = 250 *μ*m.

VEGFR-2 is thought to be the principal mediator of angiogenesis, although is also expressed at low levels in LECs. VEGFR-3 is a crucial receptor for the growth of lymphatic vessels during development and in adult life. Ligand binding promotes receptor dimerization, which may occur between two VEGFR-3 molecules (homodimerization) or between a VEGFR-3 and a VEGFR-2 molecule (heterodimerization). These biochemical events play a fundamental role in lymph-angiogenesis [36, 37]. As shown in Figures 4(a) and 4(b), both VEGFR-2 and VEGFR-3 were unambiguously expressed by human HL-LECs* in vitro*. Immunofluorescence signals of these epitopes were not detected in negative control cell lines (supplemental figure S3).

EGFR, also known as ErbB1 and HER-1, the first type I receptor tyrosine kinase identified, is a member of the ErbB, or HER, family of receptors (EGFR, HER-2, HER-3, HER-4). EGFR is a transmembrane protein with an internal tyrosine kinase domain. Activation by one of its many ligands leads to dimerization of EGFR, either with another EGFR (homodimerization) or one of the other receptors from the HER family (heterodimerization), which activates the catalytic system of the tyrosine kinase [36, 38]. This results in the activation of multiple pathways that promote survival, proliferation, and angiogenesis. These activities are mediated primarily through the mitogen-activated protein kinase (MAPK) and phosphatidylinositol 3-kinase (PI3K)-Akt pathways [38]. The typical expression of EGFR protein in primary cultures of HL-LEC is shown in Figure 4(e).

Similarly, FGFR-1 belongs to a highly conserved receptor tyrosine kinases family: FGFR1–4. This receptor activates an array of downstream signaling pathways, such as MAPK and PI3K/Akt pathways. Both these pathways regulate key cell behaviors such as survival, proliferation, differentiation, and lymphangiogenic effects in target cells [39]. As shown in Figure 4(f), virtually all HL-LECs* in vitro* express FGFR-1.

The type 1 Insulin-like Growth Factor Receptor (IGF-1R) belongs to the RTK superfamily and mediates crucial signaling pathways implicated in growth, proliferation, differentiation and apoptosis. Upon ligand binding at the plasma membrane, signaling events mediated by the IGF-1R occurs primarily through activation of PI3K-Akt and MAPK pathways [40]. HL-LECs cultured* in vitro* express IGF-1R, as shown in Figure 4(g).

The c-MET receptor tyrosine kinase, along with its ligand Hepatocyte Growth Factor (HGF), plays an important role in the regulation and control of tissue homeostasis under normal physiological conditions. However, HGF/c-MET axis has also been implicated in the regulation of cancer cell growth, angiogenesis, invasion, and metastasis. In addition to the interaction with other angiogenic signals, c-MET appears to act as an independent angiogenic factor [41, 42]. HL-LECs express c-MET receptor with its characteristic pattern (Figure 4(h)).

PDGFR-beta is a master regulator of lymphangiogenesis possessing diverse range of biologic activities, including cell survival, proliferation and chemotaxis [43]. Accordingly, we could observe that our preparations of HL-LEC widely expressed* in vitro* PDGFR-beta (Figure 4(d)).

Finally, the expression of neurotrophin receptors TrkA and p75^NTR^ were evaluated, because recent studies have shown that both receptors are involved in vascular biology. The high-affinity TrkA receptor serves as a receptor for certain neurotrophic factors, including nerve growth factor (NGF), brain-derived neurotrophic factor (BDNF), and neurotrophin 3 (NT3), and plays a critical role in the development and maintenance of the central and peripheral nervous systems. The low-affinity p75^NTR^, expressed in Schwann cells and neurons and belonging to the tumor necrosis factor family, contributes to a plethora of signaling complexes associates with several co-receptors, including the TrkA [44–47]. As shown in Figures 4(i) and 4(j), HL-LEC identified by our laboratory express TrkA and p75^NTR^.

### 3.7. *In Vitro* Angiogenesis Assay

The formation of capillary-like tubes is a rather specific functional characteristic of endothelial cells [31, 48]. To determine whether our cell population had angiogenic property at least* in vitro*, we investigated the capacity of HL-LEC to organize into tubule-like networks on Matrigel. CD31^pos^/Pdn^pos^ LECs were able to form tube-like structures in response to a matrix, which mimics the physical and functional properties of basement membrane (Supplemental data, figure S5).

## 4. Discussion

The blood vascular system has been intensively studied since its discovery, however, only in recent years, the lymphatic system has emerged as a crucial player in physiologic processes and in a variety of diverse pathologic conditions such as inflammation, wound healing, and tumour metastasis. Specifically, pulmonary lymphatic vessels are required for efficient respiration, mainly attributable to alveolar clearance in which their essential contribution has been established. Lymphatics have been also implicated in the pathogenesis of idiopathic pulmonary fibrosis, lymphangioleiomyomatosis and metastatic cancer, representing a potential novel approach to develop effective target therapies of severe lung diseases [1, 13, 17, 20].

The endothelium forms the inner cellular lining of blood and lymphatic vessels. Endothelial cell phenotypes are differentially regulated in space and time, giving rise to the phenomenon of “EC heterogeneity” implying that each vascular bed has unique structural and functional properties [49].

Cell culture models may be crucial to study the pathophysiologic role of the lung microvascular endothelium. Most of our knowledge is derived from studies on cultured human umbilical cord vein endothelial cells (HUVECs) due to their accessibility and reproducible isolation, but do not represent a model of the adult human microcirculation. Most studies have been successful in the identification and purification of blood endothelial cells from lung microcirculation. In addition, methodologies as automated cell sorting are costly, time consuming and not easily accessible to every laboratory. Moreover, only recently, lymphatic endothelial cell lines have been produced and although expensive are commercially available.

Therefore, our laboratory developed a protocol, for the routine and large scale in-house use, to harvest, isolate and expand human lung lymphatic endothelial cells.

We believe that several steps were critical to the success and effectiveness of our procedure to obtain a pure population of lymphatic endothelial cells from human lung microcirculation.

First, the fast (within one hour) processing of biological material immediately after resection ensured the preservation of cell viability and biological properties. Furthermore, daily observation of endothelial cell morphology and growth was crucial to determine the optimal time for bead separation and to avoid overgrowth of unwanted cells. Finally, cell sorting using markers such as CD31 and Pdn that are undoubtedly, abundantly, and specifically expressed by lymphatic endothelial cells both* in vivo* and* in vitro* allowed reproducible and efficient results in the isolation of HL-LEC. The immunocytochemical detection of cell surface markers confirmed that isolated cells were of lymphatic origin. These phenotypic characteristics were confirmed by flow cytometry.

The absence of *α*-smooth muscle actin was important to rule out contaminating cells in cultures immediately after the double selection for CD31 and Pdn. These data were further strengthened by the presence of lymphatic endothelium-specific markers such as Lyve-1 and Prox-1.

We also documented that our harvested and expanded HL-LEC are functionally competent. Indeed, when plated on a reconstituted basement membrane matrix, HL-LEC rapidly attached, aligned, and formed capillary-like tubules, mimicking lymphangiogenesis. Lymphangiogenesis is the formation of new lymphatic vessels from preexisting ones, an important biological process associated with diverse pathologic states. This assay is easy and cost-effective, and implies that HL-LECs are able to activate signaling pathways involved in cell adhesion, migration and protease activity ultimately leading to tubule-like microvessel formation.

To obtain insights on lymphatic endothelial cell biology, we determined whether our preparations of HL-LEC express relevant receptor tyrosine kinases (RTKs).

RTKs are central players in signal transduction networks and ligand binding activates the intrinsic protein tyrosine kinase domain triggering the intracellular signaling cascade.

We documented that virtually all HL-LECs,* in vitro*, retain the expression of VEGFR-2, VEGFR-3, EGFR-1, FGFR-1, c-Met, IGF-R1, PDGFR-beta, TrkA, and p75^NRT^. In addition to their implication in cell survival, proliferation, migration, and lympho-angiogenesis, RTKs have become the most studied class of drug target by the pharmaceutical industry.

## 5. Conclusion

We have been able to reproducibly isolate human lung lymphatic endothelial cells from healthy pulmonary tissue by double magnetic cell sorting using a combination of commercially available antibodies. The few unsuccessful attempts occurred during the initial application of our procedures and were mainly attributable to setting the time of tissue digestion and microbial contamination. Full characterization of HL-LECs confirmed the high levels of purity and successful cultures yield large number of cells that could be expanded for several passages and cryopreserved being viable for further studies.

HL-LEC may represent an important tool for* in vitro* studies on lymphatic biology, lympho-angiogenesis, wound healing, inflammation, anti-cancer therapy, and interaction with microbial agents. Our report suggests a purification strategy to isolate BECs and LECs that can be applied to other human tissues. We believe that organ- and patient-specific endothelial cells are essential to elucidate signalling pathways involved in the pathogenetic mechanisms of lung diseases and to provide novel approaches to reach the goal of a true personalized therapy.

## Supplementary Material

Supplemental figure S1. Immunohistochemical staining of human lung tissue.
Supplemental figure S2. Immunocytochemical analysis of HL-BEC.
Supplemental figure S3. Negative controls for immunofluorescence staining.
Supplemental figure S4. Immunocytochemical staining for CD45.
Supplemental figure S5. Tubulogenesis assay.
