# Supplementary material for: Isolation and Characterization of Human Lung Lymphatic Endothelial Cells
Source: Biomed Res Int. 2015 Jun 7;2015:747864. doi: 10.1155/2015/747864 (PMC4475539; doi:10.1155/2015/747864)
Supplement: Supplementary file 1 — Supplemental figure S1. Immunohistochemical staining of human lung tissue. Supplemental figure S2. Immunocytochemical analysis of HL-BEC. Supplemental figure S3. Negative controls for immunofluorescence staining. Supplemental figure S4. Immunocytochemical staining for CD45. Supplemental figure S5. Tubulogenesis assay. [file 747864.f1.pdf]

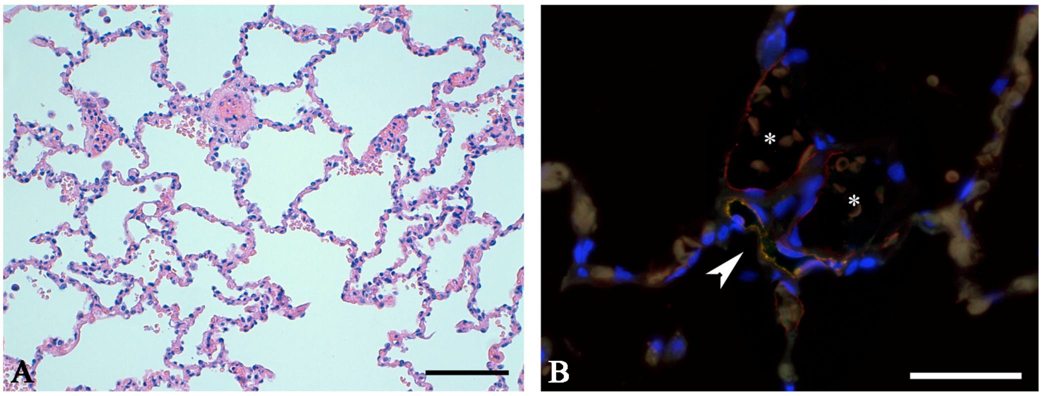

Supplemental figure S1. A: H&E stained section of the alveolar parenchyma of the normal human lung. B: section of the normal human lung subjected to double immunofluorescence staining for CD31 (red) and Podoplanin (Pdn, green). Arrow points to a lymphatic vessel documented by yellow fluorescence as a result of the combination of the red fluorescence of CD31 and the green fluorescence of Pdn immunolabelling. Blood vascular lumens (*) containing red blood cells are depicted by CD31 only while the Pdn^{pos} lymphatic is devoid of erythrocytes. Nuclei are stained by the blue fluorescence of DAPI. Scale bars: A= 100μm; B= 50μm.

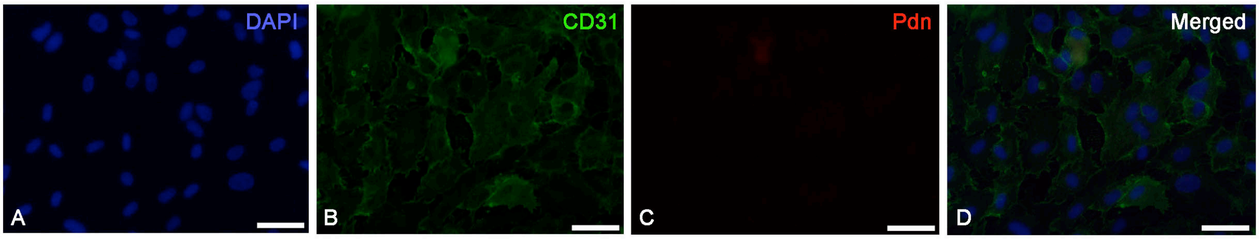

Supplemental figure S2. Immunocytochemical analysis of HL-BEC. Human lung BECs express the surface antigen CD31 (B, green fluorescence). whereas are negative for Podoplanin (Pdn) documenting the absence of contaminating LECs. Nuclei are stained by the blue fluorescence of DAPI. Scale bars: A-D= 50μm

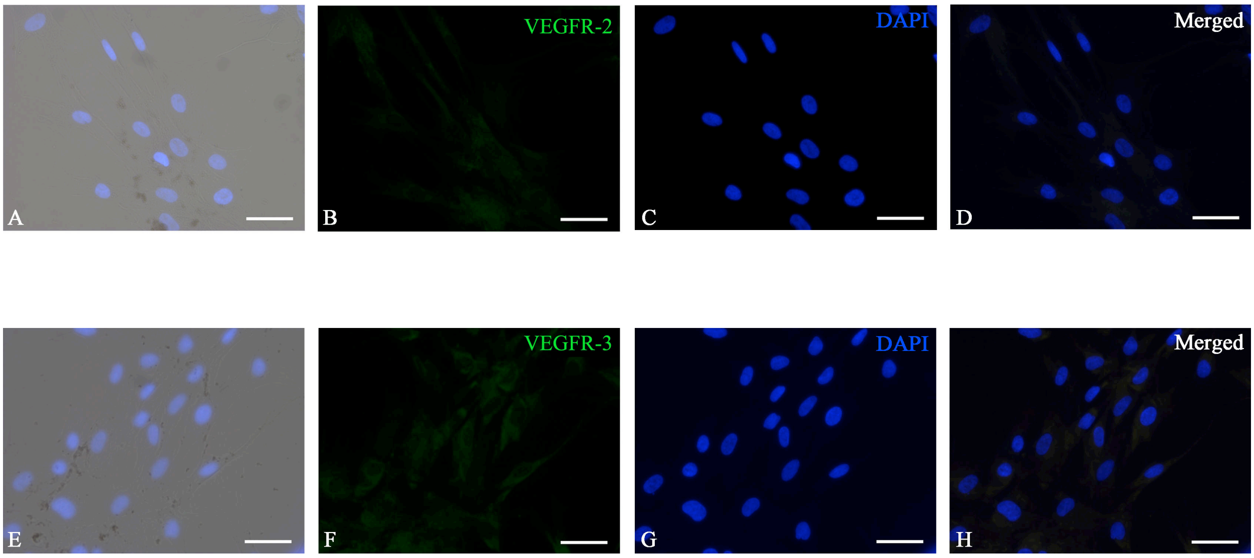

Supplemental figure S3. Immunofluorescence staining of VEGFR-2 (upper panels) and VEGFR-3 (lower panels) in cultured human dermal fibroblasts used as negative controls. A and E correspond to the combination of images taken by phase contrast and DAPI staining of nuclei. B and F show a slight background green fluorescence of VEGFR-2 and VEGFR-3 antibodies revealed by FITC-conjugated secondary antibodies. Nuclei only are shown by the blue fluorescence of DAPI in C and G. D and H: images merged from B- C and F-G, respectively. Scale Bars: A-H= 50 μm

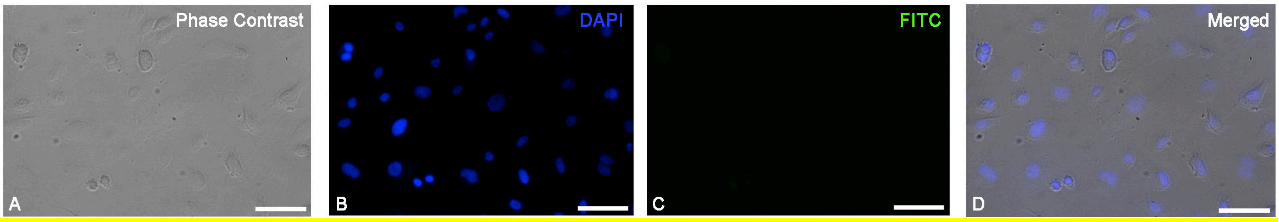

Supplemental figure S4. Immunostaining of HL-LECs incubated with CD45 as a negative control isotype primary antibody. (Scale bars: A-D= 75µm

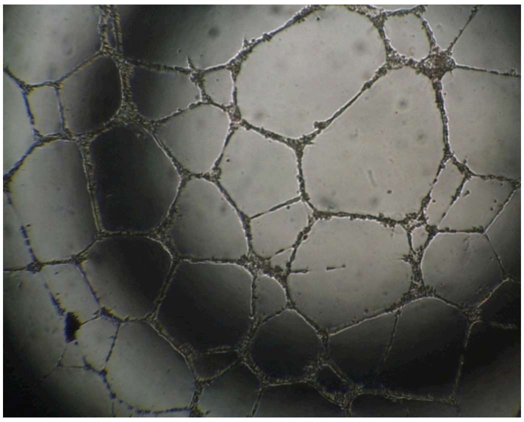

Supplemental figure S5. Tubulogenesis assay. A representative phase contrast image acquired 24 hours after plating $2x10^4$ HL-LECs per well documenting the ability of HL-LEC to form tubular structures when seeded on Matrigel®.
